# Impacts of the Early Collaborative Intervention on mother-preterm infant interaction at one month of age: Secondary analysis of a randomized controlled trial

**DOI:** 10.1016/j.ijnsa.2026.100507

**Published:** 2026-02-09

**Authors:** Charlotte Sahlén Helmer, Ulrika Birberg Thornberg, Thomas Abrahamsson, Evalotte Mörelius

**Affiliations:** a Department of Health, Medicine and Caring Sciences, Division of Nursing Sciences and Reproductive Health, Linköping University, Linköping, Sweden; b Crown Princess Victoria Children ´s Hospital, Linköping University Hospital, Linköping, Sweden; c Department of Rehabilitation Medicine, and Department of Health, Medicine and Caring Sciences, Linköping University, Linköping, Sweden; d Department of Behavioural Sciences and Learning, Linköping University, Linköping, Sweden; e Department of Biomedical and Clinical Sciences, Division of Children's and Women's Health Linköping University, Linköping, Sweden; f School of Nursing and Midwifery, Edith Cowan University, Joondalup, Western Australia, Australia

**Keywords:** Early intervention, Health care quality, access, and evaluation, Infant-mother interaction, Neonate, Nursing research, Preterm infant, Relations

## Abstract

**Background:**

A sensitive, well-functioning maternal interaction is vital for healthy infant development. For moderate to late preterm infants, this is even more important as this group of infants are at increased risk of facing neurodevelopmental disorders. The Early Collaborative Intervention supports parent-preterm infant interaction and includes three sessions, two in the hospital and one after discharge.

**Objective:**

To investigate the impact of the Early Collaborative Intervention, compared with standard care, on mother-preterm infant interaction at one month corrected age.

**Design:**

A longitudinal randomized controlled trial, reporting secondary outcomes from the first follow-up.

**Setting:**

The intervention was conducted at a pediatric center with two neonatal intensive care units with an infant and family centered approach. The intervention was evaluated in the homes of the families.

**Participants:**

Families with preterm infants born in gestational week 30+0–35+6 (*n* = 143) were randomized. In this one-month follow up a total of 101 families participated, (standard care with the Early Collaborative Intervention, *n* = 60, standard care, *n* = 41).

**Methods:**

The mother-infant interactive behavior was videotaped during a bath and later analyzed with Ainsworth’s Maternal Sensitivity Scales and the Emotional Availability Scales. The coder was masked to group randomization as well as to demographic data of the dyads.

**Results:**

In the analysis the maternal mean scores were statistically significantly higher for the intervention-group versus the standard care group in the Availability subscale, 7.30 vs 6.29 (CI 0.01–0.86, *p* = 0.045, Cohen’s d 0.43), and Acceptance subscale, 8.00 vs 7.22 (CI 0.12–0.97, *p* = 0.012, Cohen’s d 0.55), in the Ainsworth’s Maternal Sensitivity Scales. Mean score were also statistically significantly higher for the intervention-group versus the standard care group in the Non-hostility subscale, 6.60 vs 6.12 (CI 0.11–0.97, *p* = 0.013, Cohen’s d 0.54), in the Emotional Availability Scales. The results suggest that these aspects of maternal interactive behavior towards her infant, are the ones most influenced by the Early Collaborative Intervention.

**Conclusions:**

The Early Collaborative Intervention had beneficial impacts on maternal interactive behavior for those who took part in three sessions or more of the intervention program.

**Registration:**

The project was registered in ClinicalTrials.gov with the number: NCT02034617, registered 19/12/2013, date of the first recruitment 15/01/2014.

**What is already known**

• Moderate and late preterm infants are a large and growing group of preterm infants

• Early interventions can improve family well-being and infant developmental outcome

What this paper adds

• The intervention had a beneficial effect on mother-infant interactive behavior

• Three sessions were needed to make a difference in maternal interactive behavior

Alt-text: Unlabelled box dummy alt text

## 1 Introduction

Maternal sensitivity was by Mary Ainsworth conceptualized as the capacity to perceive and interpret infant signals and to respond both promptly and appropriately (Ainsworth et al., 1978). A well-functioning mother-infant interaction has been described as a rhythmic, dyadic act (Brazelton et al., 1975). A sensitive maternal interactive behavior lays the foundation for secure infant attachment, and for infant emotional and cognitive development (McMahon et al., 2022; Miller et al., 2022; Treyvaud et al., 2016). Furthermore, a sensitive interactive maternal behavior positively affects language and social development in children (Deans, 2020). A high quality of maternal interaction has been shown to mitigate the risk of preterm birth on infant cognitive outcome, suggesting that a well-functioning interaction pattern may act as a protector in a vulnerable infant population (Hall et al., 2015a; Poehlmann et al., 2001).

## 2 Background

Preterm birth is often an unexpected event, with a risk of having a negative impact on parent-infant interaction because of the environment in the neonatal intensive care unit, including separation. Admission to a neonatal unit puts parents in a fragile position (Carton et al., 2020; Ionio et al., 2019) and stressful parental experiences may further compromise the quality of parent–infant interaction (Bilgin et al., 2015; Hamon et al., 2023). Mothers of preterm infants often experience a higher degree of physical illness during pregnancy and childbirth (Goldenberg et al., 2008) including medical treatment that may limit their ability to establish physical closeness with their newborn. Furthermore, preterm infants exhibit more subtle behavioral cues, making them more difficult to detect and respond to (Feldman et al., 2006). They demonstrate lower levels of physiological and emotional regulation, and heightened reactivity to environmental disturbances compared to full-term infants (Feldman, 2006). Taken together, these factors compromise mother–infant interaction. Increasing evidence is emerging of the importance of including both parents in care and interventions for optimal infant development, as they are both part of the interactional pattern in the family (Filippa et al., 2021; Mascheroni et al., 2019).

Infants born late preterm are at risk of facing neurodevelopmental disorders (Cheong et al., 2017; Crump et al., 2021; Martinez-Nadal et al., 2021; You et al., 2019). They have an immature neurological system (Feldman, 2006) and the time after birth is sensitive where infant environmental experiences affect cognitive and emotional development (Krugers et al., 2016). Infants born preterm between gestational week 30 and 36 usually have a shorter hospital stay than preterm infants born before gestational week 30. For optimal infant development and the wellbeing of the family, it is important that care is tailored to support the individual needs of both the infant and the parents, and that care prepares the family for discharge from the hospital. However, as moderate to late preterm infants is a large and growing group of infants due to maternal factors like older age and multiple birth (Karnati et al., 2020), it may be difficult to ensure individualized support in a busy neonatal intensive care unit.

As early experiences impact infant future development (Britto et al., 2017), different interventions have been designed to support parents and preterm infants (Puthussery et al., 2018). Interventions with a clear focus on improving parental sensitive behavior have been shown to be more effective than interventions having a broader aim (Bakermans-Kranenburg et al., 2003). The Early Collaborative Intervention was considered to fill a gap as it was developed specifically to suit moderate to late preterm infants and their parents. The intervention initiates support for interaction already within the hospital setting. The support is fostering parent–infant interaction by equipping parents at an early stage with strategies to recognize, interpret, and appropriately respond to the infant’s subtle cues and signals of contact. The developmental framework and practical provision in detail have previously been reported (Helmer et al., 2021) as have the mothers’ experiences of the intervention program (Helmer et al., 2022). The primary outcome of the Early Collaborative intervention-trial measured with the Bayley-III when infants were one year of age, demonstrated statistically significantly improvements in communicative development in favor for the intervention compared to standard care (Birberg Thornberg et al., 2025).

## 3 Study aim and hypothesis

The aim of this study was to investigate the impact of the Early Collaborative Intervention, compared with standard care, on mother-preterm infant interaction at one month corrected age. Our hypothesis was that the Early Collaborative Intervention would benefit mother-infant interaction compared with standard care.

## 4 Methods

### 4.1 Study design

The two-armed randomized controlled trial has a longitudinal design. This study reports secondary outcomes at the first follow-up at one month corrected age. The study was registered the 19/12/2013 on ClinicalTrials.gov (NCT02034617), https://clinicaltrials.gov/search?term=NCT02034617.

### 4.2 Participants and setting

Inclusion criteria were families with infants born between gestational week 30+0 days and 35+6 days. The parents had to understand and write Swedish as the questionnaires used at inclusion and follow-ups were in Swedish. Exclusion criteria were infant congenital major malformations, complications known to affect infant cognition, as primary outcome of the project was infant cognition at one year of age. Families living outside the county of Östergötland, Sweden were excluded as follow-up assessments were conducted in the families’ homes, and the geographical distance to the families imposed practical limitations.

The setting was a pediatric center with two neonatal intensive care units, one Level II and one Level III, with a total of 24 cots. International standards describe Level II as a unit caring for preterm infants born after gestational week 32, a Level III unit cares for infants born after gestational week 22 (Stark, et al., 2023).

### 4.3 Standard care and the early collaborative intervention

The control group in this study received standard care. Standard care in the neonatal intensive care units included a child and family centered approach with single-family rooms. Parents and siblings could stay at the unit 24/7. Families had a kitchen area where they could prepare and eat meals and socialize with friends or relatives. Early and regular skin to skin contact was part of the care. Infants born preterm after gestational week 30 were often cared for in family rooms. The Newborn Individualized Developmental Care and Assessment Program (Als, 1986) was praxis; the parents were the primary care givers and took an active part in medical rounds. Care of infants born moderate to late preterm and their parents was given in a formal manner, with no extra structural support to parents in terms of facilitating parent-infant interaction. When the preterm infant could feed small amounts and achieved cardiorespiratory stability, the family was discharged to homecare. During homecare, there were checkups often twice a week, sometimes by a telephone or during a web-based meeting. When the infant no longer needed a feeding tube for supplementary feeding, the family was discharged.

**Fig. 1:**
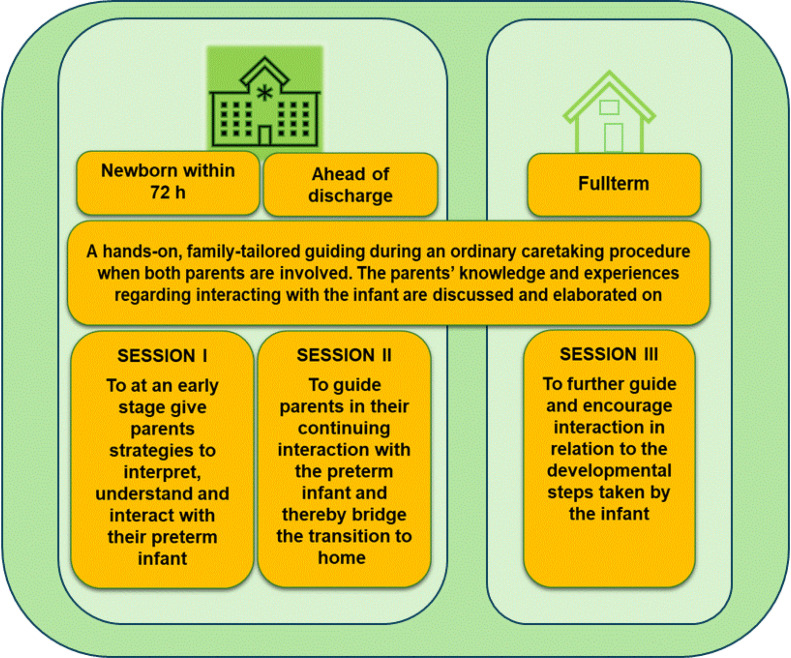
The Early Collaborative Intervention program depicted (Birberg Thornberg et al., 2025).

The group randomized to intervention received Early Collaborative Intervention additional to standard care (Helmer et al., 2021). The first two sessions were provided in the single-family room in the neonatal intensive care unit. Additional sessions could be provided between the first and the second at the hospital, depending on the length of the family’s hospital stay and the needs of the family. The intervention was provided individually for each family, with both parents present and at a time that suited the preterm infant and its parents. The Early Collaborative Intervention session was tailored to the family's specific needs, building on the preterm infant’s interactive behavior and the parents' knowledge, immediate experiences and emotions. During a session parents were verbally and hands-on guided to instantly respond to their infant’s cues of communication in a suitable way for the infant. The session was provided during care practice, for example during a diaper change or breastfeeding. During this first session, within 72 h after birth, parents were guided in how to interact with their infant at an early stage and the parents’ early experiences were discussed and elaborated on. Ahead of discharge, a second session was provided for the family, this time focusing on how the parents could interact with and thereby support their infant at home. When the infant was full-term and discharged from neonatal homecare, a final session was provided at the family’s home. During this session, the infant’s interactive developmental progress was discussed, and the parents were guided in the future needs of the infant. Each session lasted for approximately one-two hours depending on the needs of the family. One target during the intervention was to empower parents and to strengthen parent-infant emotional mutuality. Another target was to raise awareness of how a sensitive parental interaction supports infant interaction. After a session, the family was given a short-written summary often including photos of the parent-infant interaction, Fig. 1.

The research core group who developed the Early Collaborative Intervention, also created a manual for the providers. To further ensure fidelity the provider-group had repeated discussions regarding the executions of the sessions (Helmer et al., 2021). The providers in this study were certified in the Newborn Individualized Developmental Care and Assessment Program (Als, 1986) to ensure they were knowledgeable about preterm infant behavior.

### 4.4 Procedure

#### 4.4.1 Inclusion procedure

Families were recruited between January 2014 and September 2020 and randomized within 72 h from birth of the preterm born infant, to receive the Early Collaborative Intervention or standard care. Sampling was purposive and consecutive, and the parents were provided with sealed envelopes for complete randomization. Once the parents had consented to the study, the families randomized to the Early Collaborative Intervention had a plan made up to have the sessions as scheduled by the study protocol. Six providers, nurses and assistant nurses trained in the Early Collaborative Intervention, were available to do the sessions, one provider for each session. The choice of intervention provider for each family depended on the work schedule of the providers. Thus, the same family had different providers across the three sessions.

A coordinator nurse at one of the two neonatal intensive care units organized the three sessions for the families included in the study. The trial concluded once the number of enrolled families met the requirement determined by the power analysis.

#### 4.4.2 Follow-up procedure

The follow-up data collection was between February 2014 and October 2020. The outcome of this study was the impact of the intervention on mother-infant interaction. This was assessed at standardized, scheduled home visit when the infant was one month corrected age. A bathing session was videotaped, where the mothers undressed their infants, gave them a bath, and dressed them again. The mothers were asked to bathe the infant as they normally would. The bathing session captured interactive behavior in a natural context. At this follow-up the mothers answered questions on breastfeeding, bath-routines, and depressive syptoms. Follow-up assessors were masked for group allocation as well as to demographic data on the dyads.

### 4.5 Ethical considerations

The study was performed in accordance with the declaration of Helsinki (World Medical Association Declaration of Helsinki, 2022). Ethical approval was obtained from the Regional Research Committee, registration number 2013/367/31. Parents received written and oral information about the study and were given opportunities to ask questions. The parents signed an informed consent form prior to becoming involved in the study.

### 4.6 Outcome measurements

#### 4.6.1 Descriptive data

Data on the infants’ gender, post menstrual age at birth, birth weight, occurrence of small for gestational age, method of delivery, respiratory support, Apgar score, phototherapy, antibiotics, and which hospital the infant was cared for at, were collected from the infants’ medical charts. The mothers responded to questions about their marital status, age, educational level, and illness as well as the number of infants, siblings, and feeding status of the infant.

#### 4.6.2 Interaction and infant behavior instruments

The videotaped bathing sessions were coded for mother-infant interaction by the first author using the Ainsworth’s Maternal Sensitivity Scales (Ainsworth, 1969), and the Emotional Availability Scales (Biringen, 2022), further described below. Cases in which scoring were unclear, were discussed between the first and last author.

##### 4.6.2.1 Ainsworth’s Maternal Sensitivity Scales

The Ainsworth’s Maternal Sensitivity Scales consists of four scales, in which four dimensions of maternal interactive behavior are assessed, each scored from one to nine (Ainsworth, 1969). The first scale reflects the mother’s sensitivity, her capability to comprehend and interpret infant interaction, and to respond to infant interactive behavior in a suitable way. The second scale, maternal cooperation, reflects the mother’s ability to adjust her interactive behavior to match the infant’s state and mood. The third considers the extent to which the mother is physically and psychologically available to the infant. The fourth scale assesses maternal acceptance of infant behavior. Typically, well-functioning mother-infant dyads score above six points (Ainsworth, 1969). The instrument has been tested (Cronbach’s alpha, 0.81) and used in various contexts (Tryphonopoulos et al., 2016). It is still used (Helmer et al., 2020; Jonas et al., 2015) and has inspired other interaction instruments (Mesman, 2013).

##### 4.6.2.2 Emotional Availability Scales

The Emotional Availability Scales consists of six scales, in which six dimensions of emotional availability between child and caregiver are assessed to capture parental and child affect and behavior. Each scale has a direct score from one to seven points (Biringen, 2022). A sensitivity score of 5.5 and above is an emotionally available interactive behavior. The first scale, adult sensitivity, reflects the parent’s ability to accurately read and respond to infant communication. Focus is on the parent’s ability to be warm and emotionally connected to the infant. The second scale, adult structuring reflects the parent’s ability to guide and support the infant. The third, adult non-intrusiveness, refers to whether the parent is available to the infant without being commanding or domineering. The fourth, adult non-hostility, refers to the parent’s ability to continuously adopt a gentle, well-regulated tone throughout the interaction. There are two scales for child interaction, where the first scale reflects the infant’s responsive behavior to parental interaction, whereas the second child scale assesses how and to what extent the infant is involving the parent (Biringen, 2022). The first and the fourth author are certified and reliable coders trained by Zeynep Biringen (Biringen, 2008). The first author was recertified in 2022. The scales are widely used and have been successfully tested (Cronbach’s alpha, 0.71- 0.84) in various contexts and cultures (Biringen et al., 2014).

#### 4.6.3 Parental self-assessed instruments

##### 4.6.3.1 Edinburgh Postnatal Depression Scale

To control for potential mother postnatal depression, the self-administered, tested (Cronbach’s alpha, 0.87) Edinburgh Postnatal Depression Scale was used (Cox et al., 1987). It evaluates feelings of sadness and unhappiness and was completed when the preterm infant was one month corrected age. It is scored on a 10-item scale, each scale ranging from 0 to 3, with a total score of 0 to 30. The Swedish versions use a cut-off for depression symptoms of 12 points (Wickberg et al., 1996).

### 4.7 Statistical methods

A power analysis prior commencement was calculated on infant cognition at 12 months, which was the primary outcome of the larger project. The sample size was calculated based on the expected difference of 0.5 SD between the intervention group and the control group in the cognitive score. A sample size of 63 infants in each group was calculated to get a statistical power of 80 %, and a significance level of 0.05 (two-tailed test). To compensate for attrition, 17 (12 %) additional families were enrolled in the study. The final sample included 143 families, (*n* = 71 in the intervention group, *n* = 72, in the standard care group).

Primary outcome variables on maternal and infant interaction were analyzed using independent sample Student’s *t*-test. Effect size was calculated with Cohen’s d on interaction variables. Cohen’s d is commonly interpreted as small *d* = 0.2, medium *d* = 0.5 and large *d* = 0.8 (Cohen, 1962). To analyze possible differences between groups related to infants’ and mothers’ background characteristics, the independent sample Student’s *t*-test was used for continuous outcome variables. Chi-2 statistics was used for categorical outcome variables.

The Early Collaborative Intervention group was defined as having all three stipulated sessions or more. Families who took part in less than three sessions were excluded in the per-protocol analysis. As there was a statistically significant difference in birthweight between groups in the analysis, ANCOVA was used with the statistically significant interaction-variables analyzed as dependent variables. The IBM SPSS statistical package software version 29 was used to analyze data.

## 5 Results

**Fig. 2:**
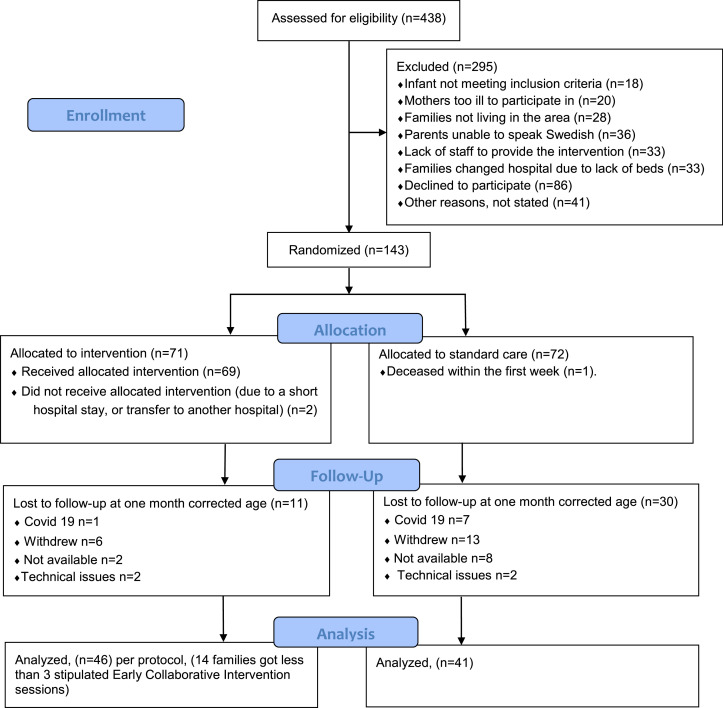
Flowchart for the sample at the first follow-up.

In this study of secondary outcomes measured at the first follow-up on mother-infant interaction, 87 families participated, 46 families in the standard care with intervention-group, 41 in the standard care-group. The participation and reasons for drop-out is described in Fig. 2.

### 5.1 Compliance with protocol

Fourteen families were not given the stipulated minimum of three intervention sessions, due to lack of providers (*n* = 2), a short hospital stay (*n* = 8), or transfer to another hospital (*n* = 4). These families were only included in the intention to treat analysis, which is reported in the supplement (Supplemental Table 1).

### 5.2 Analysis

Forty-six families from the intervention group were included in the per-protocol analysis. Out of these 46 families, 40 (87 %) were given three sessions, four families had four sessions, and two families were given five sessions.

#### 5.2.1 Participants’ characteristics

In the analysis the mean birthweight for the intervention group was statistically significantly lower than for the standard care group. No other statistically significant demographic differences between the groups appeared (Table 1). As infant birthweight was statistically significantly different between the two groups, ANCOVA was calculated using infant birthweight as a covariate and the interaction-scales with statistically significant differences between groups as dependent variables. However, birthweight did not change the results, i.e. ANCOVA analyses confirmed analyses made with Student’s *t*-test.

**Table 1:** **Demographic data and characteristics** of participating infants and mothers.

	Early Collaborate Intervention *N* = 46	Standard care *N* = 41	p-value
*Infant demographics/characteristics*			
Gender girls/boys	23/23	16/25	0.30
Twins ( %)	5 (10.8)	2 (4.9)	0.31
Gestational week at birth, mean week+days (SD)	33+3 (1.67)	33+6 (1.39)	0.12
Birthweight gram, mean (SD)	2187 (463)	2416 (570)	0.04
Small for gestational age ( %)	9 (19.5)	4 (9.8)	0.20
Vaginally delivery, numbers ( %)	33 (71.7)	29 (70.7)	0.92
Siblings numbers ( %) /missing data	18 (40.9) /2	21 (51.2) /1	0.29
Respiratory support first week, ventilator, CPAP, high flow, numbers ( %)	13 (28.2)	14 (34.1)	0.55
Apgar score 5 min, mean (SD)	9.30 (1.25)	9.29 (1.18)	0.97
Phototherapy, numbers ( %)	31 (67.3)	22 (53.7)	0.19
Antibiotics during NICU stay, numbers ( %)	5 (10.8)	6 (14,6)	0.60
Exclusively breastfed at 1 month, numbers ( %)	29 (63.0)	21 (51.2)	0.73
Bathing frequency per week, less than once/ once a week/more than once a week/missing	2/26/12/6	0/22/10/9	0.44
If the infant likes to bath/never likes/missing	38/2/6	32/0/9	0.20
Level 3 NICU/level 2 NICU/both	40/4/2	36/4/1	0.55
*Maternal demographics/characteristics*			
Single parent	1	1	Not computed
Mother´s age years, mean (SD)	31 (5.80)	31 (5.45)	0.71
Maternal educational level, elementary school/high school/ university/ other/ missing	0/13/29/3/1	1/13/21/2/4	0.63
Maternal chronic illness, numbers ( %)	8 (17.3)	10 (24.4)	0.45
Edinburgh Postnatal Depression Scale, mean (SD) / missing numbers, over cut off ( %)	6.16 (5.32) /8, 5 (10.9)	5.12 (5.00) /7, 3 (7.3)	0.40

CPAP= Continuous Positive Airway Pressure, NICU= Neonatal Intensive Care Unit.

Student’s *t*-test was used to compare means. Chi-2 statistics was used for categorical outcome variables and frequencies.

#### 5.2.2 Effects of the early collaborative intervention on Ainsworth’s Maternal Sensitivity Scales

In the Ainsworth’s Maternal Sensitivity Scales, the mean scores were statistically significantly different between groups in the Availability and Acceptance subscales. The effect size measured with Cohen’s d, was small to medium (Table 2).

**Table 2:** Videotaped interaction scored with **Ainsworth***’***s Maternal Sensitivity Scales and Emotional Availability Scale direct score**, in dyads included in the per protocol analysis.

	Early Collaborative Intervention *n* = 46	Standard care *n* = 41	P-value*	Cohen*’*s d	Confidence Interval 95 % for Cohen*’*s d	
					Lower	Upper
*Ainsworth’s Maternal Sensitivity Scales, mean (SD)*						
Sensitivity	6.50, (2.06)	5.78, (2.30)	0.127	.331	.094	.754
Cooperation	6.46, (1.74)	5.68, (2.13)	0.065	.401	.025	.825
Availability	7.30, (2.06)	6.29, (2.56)	0.045	.438	.010	.862
Acceptance	8.00, (1.25)	7.22, (1.59)	0.012	.550	.120	.978
***Emotional Availability Scales,mean (SD)***						
Sensitivity	5.25, (1.42)	4.89, (1.51)	0.256	.245	.178	.667
Structuring	4.95, (1.68)	4.50, (1.62)	0.213	.269	.154	.692
Non-intrusiveness	4.86, (1.32)	4.43, (1.48)	0.153	.309	.115	.732
Non-hostility	6.60, (0.74)	6.12, (0.99)	0.013	.548	.117	.975
Child responsiveness	4.55, (1.60)	4.41, (1.66)	0.691	.086	−0.336	.507
Child involvement	4.16, (1.55)	4.06, (1.74)	0.773	.062	−0.359	.483

⁎ *Student’s t-test was used to compare means.*

#### 5.2.3 Effects of the early collaborative intervention on Emotional Availability Scales

In the Emotional Availability Scales, the mean direct score was statistically significantly different between groups in the subscale Non-hostility, with a medium effect size measured with Cohen’s d (Table 2).

### 5.3 Drop out analyses

Demographic data of infants and mothers lost at follow-up was analyzed (in total *n* = 42) and compared with participants completing follow-up. There was a statistically significant difference in maternal illness between the groups, where more mothers lost at follow-up had some sort of chronic disease (Supplemental Table 2).

## 6 Discussion

Our hypothesis that the Early Collaborative Intervention was superior to standard care in improving maternal-infant interaction at one month corrected age was supported. The differences were statistically significant in the Ainsworth’s Availability subscale, with mothers being more aware of her infant’s signals and communication and more accessible psychologically, and in the Acceptance subscale, where mothers were more tolerant and patient. Moreover, in the Non-hostility subscale in the Emotional Availability Scales, mothers displayed less hostile behavior. Their behavior was gentler, and less boredom and discontent were shown. The largest effect sizes were found in the Acceptance and Non-hostility subscales.

Availability is an area of focus in the Early Collaborative Intervention. Thus, it is not surprising that availability improved in the intervention group. This is also in line with the findings from our qualitative study in which one mother expressed her experiences thus: *“He was just a baby that one didn't really know, with the feedback from the interventions, then it becomes more like, yes, this little person, he wants it this way and so we need to be more observant at he like wants it in this or that way… we thought that he was just like this is a baby, a little bit like that, without that Henrik is an individual person”, p. 2896* (Helmer et al., 2022).

Mothers in the intervention group were more accepting towards their infant, showing a more tolerable interactive behavior even when the infant’s behavior was less desirable. Their interaction with the infant was more respectful, and they showed more positive feelings towards their infant. This concurs with a study evaluating a more extensive group intervention for mothers in need of extra support which found statistically significant differences post intervention in maternal acceptance, however, like in our study, not in maternal sensitivity (Zeegers et al., 2019).

The non-hostility-effect is in line with a study of full-term at-risk dyads that compared interaction with the Emotional Availability Scales after a three-session intervention based on the Newborn Behavioral Observations (Nicolson et al., 2022). In the Non-hostility sub-scale mothers in both groups generally scored high. For example, hostile play that is scored in this item, was not exhibited by any of the mothers and holds limited relevance in this context. However, more mothers in the standard care group displayed covert hostility like a lack of interest in the infant and disappointment at the infant’s behavior. This may be attributable to a similar mechanism as observed with Ainsworth’s Maternal Sensitivity Acceptance subscale, whereby increased knowledge of infant communication facilitates greater interest in and satisfaction with the infant’s actions and awareness of the importance of one owns behavior towards the infant.

These beneficial impacts on maternal interactive behavior may have contributed to the observed positive outcomes on the child’s language development at one year of age (Birberg Thornberg et al., 2025). This is particularly important, as the Early Collaborative Intervention- by providing early support for communicative interaction- may help mitigate the well-documented adverse effects of preterm birth on linguistic development (van Noort-van der Spek et al., 2012).

The effect sizes were larger in the Ainsworth’s Maternal Sensitivity Scales as compared to the Emotional Availability Scales. This may indicate that the Ainsworth’s Maternal Sensitivity Scales more easily captures differences in interaction behavior in this population. The scales were designed to evaluate interaction in infancy (Ainsworth et al., 1974), whereas the Emotional Availability Scales infancy/early child version are made for dyads with children up to 5 years of age (Biringen, 2022). Although both scales measures maternal sensitivity, the perspective on sensitivity is a bit different in the different scales and they are not interchangeable as they capture distinct dimensions in relation to infant behavior (Bohr et al., 2018). Both scales have been tested in different contexts (Tryphonopoulos et al., 2016), however, there may be aspects in the dyadic interactive behavior that is more easily captured with the Ainsworth’s Maternal Sensitivity Scales.

Larger effect sizes have been shown in studies with more vulnerable samples (Nowak et al., 2008) as well as in families expressing more distress pre-intervention (Chamberlain et al., 2008). In the present study we included families regardless of psychosocial vulnerability or health issues, still we found medium effect sizes. The population was relatively well-educated and moreover, there were no statistically significant differences in maternal education level, maternal illness or in the Edinburgh Postnatal Depression Scale between groups.

Infants in the intervention group had a lower mean birthweight than infants in the standard care group. According to Feldman and co-workers, infants with lower birthweight are less robust and have weaker signals for interacting (Feldman, 2006; Feldman et al., 2006). The fewer the prominent interaction signals, the more difficult for parents to see, interpret and respond to their infants (Feldman et al., 2007; Harel et al., 2011; Hawthorne, 2005). Our results indicate that mothers in this group benefitted from this type of intervention and received higher mean-scores for interaction, which further supports the notion that the intervention has been beneficial.

The Early Collaborative Intervention is a bidirectional intervention for supporting, educating and guiding parents while the infant is simultaneously supported and comforted by the parents. The intervention successfully benefited maternal interactive behavior but no differences regarding infant interaction behavior was found in this follow-up. Kalinauskiene et al. (2009) showed similar results where mothers seemed to benefit more from the intervention than infants did (Kalinauskiene et al., 2009). The time point chosen for assessing these variables may be crucial. Infant involvement measured by the Emotionally Availability Scales has been found to correlate with age; the older the child the easier to involve the parent (McConnell et al., 2020). Moreover, preterm birth affects the infants’ behavior repertoire (Hall et al., 2015b). This preterm population may need more time to be able fully to express responsiveness and involvement as measured in the Emotional Availability Scales (Stack et al., 2018).

An incidental effect from the Early Collaborative Intervention may involve staff being willing to listen actively, and to collaborate and support parent-infant interaction. Having time to spend with one family at a time engenders a trustful staff-parent relationship, which is needed for mutual reflections. This has been described as important by staff (Franck et al., 2022) and parents (Øberg et al., 2023). Moreover, it was highlighted by the mothers when they shared their experiences of the Early Collaborative Intervention (Helmer et al., 2022). Interventions that support positive interactions between parents and their preterm infants typically begin in the hospital and extend into the home environment (Puthussery et al., 2018). These interventions often focus on enhancing parental responsiveness and providing structured guidance in caregiving (Benzies et al., 2013). Such strategies form essential components of the Early Collaborative Intervention, explaining why the mothers in the intervention group are more sensitive and attuned in their interaction behavior.

### 6.1 Clinical implication

The findings imply that support and guidance on interaction makes a difference. As described by Ainsworth (Ainsworth, 1969) and Biringen (Biringen, 2022) even minor differences in scorings may have a major clinical impact for the infant. Therefore, even small effect sizes can have a major impact depending on the context in which they are found in (Lakens, 2013). Thus, it is important to support the interactive behavior of parents in a vulnerable period of infant development (Bilgin et al., 2015). This study demonstrates that an early intervention consisting of only three sessions has the potential to influence the early mother–infant interaction.

### 6.2 Strengths and limitations

One strength of this study is the relatively large sample size, which is comparable to other studies on maternal sensitivity towards preterm infants (Bilgin et al., 2015).

A systematic review and meta-analysis targeting full-term at-risk and preterm infants showed how early interventions for improving parental interactive behavior, were able to affect infant well-being and development, and that the effects were larger in low- and middle-income countries (Jeong et al., 2021). The present study adds to this by showing the positive impact of an early intervention in a high-income country, with good social security, including paid parental leave and an opportunity for both parents to be present 24 h a day.

The instruments used for evaluation in this study are robust and widely used (Tryphonopoulos et al., 2016). This study contributes to the knowledge of the use of the Emotional Availability Scales by being the first study to evaluate mother-preterm infant interaction at this early stage compared to previous studies (McMahon et al., 2020, 2022; Salvatori et al., 2016; Stack et al., 2018).

This study has certain limitations that should be considered when interpreting the results. The standard care in the present study might not be representative due to the high standard of the neonatal units included. The units have a long tradition of educating and certifying staff in the Newborn Individualized Developmental Care and Assessment Program (Als, 1986) as well as caring for infants in a suitable developmentally supportive manner. Furthermore, all the infants are cared for in separate rooms where both parents can be present 24 hour a day with paid parental leave. This high standard setting may have reduced the differences between the groups.

The families could not be masked as this might have influenced intervention efficiency. However, the follow-up evaluation was masked. Fathers were included when provided with the intervention as both parents are important for infant well-being and development (McMahon et al., 2019). However, the fathers have not been included in the interaction evaluation as we could not demand that both parents were present at the follow-up. An evaluation of the fathers’ interactive behavior would have been favorable.

Drop-outs amounted to 42 families in the study. During parts of the study time, the Covid-19 pandemic prevented follow-up visits and affected the total drop-out rate. Moreover, a higher proportion of mothers among the drop-outs had a chronic disease, which may have affected their ability to attend the follow-up. Fewer participants in the intervention group were lost to follow-up, in line with other studies in the field (Chiu et al., 2009; McMahon et al., 2019; Stack et al., 2018).

Among families assessed for eligibility in this neonatal study, some (*n* = 86) declined participation, in line with a previous study (Mörelius et al., 2020). The stress of giving birth to a preterm infant, and the overwhelming situation parents found themselves in might be a reason for this. As we do not have demographic data on parents opting out, we do not know whether and how it may have affected our results.

Since it was not possible to include a baseline assessment, we have not been able to control for mother-infant interaction style at birth.

## 7 Conclusion

The Early Collaborative Intervention yielded beneficial outcomes on maternal-infant interaction compared to standard care at one month corrected age, despite being a short three-session intervention. The results are encouraging as one of the targets of the Early Collaborative Intervention is to support parents’ awareness of the preterm infants’ communication directly after birth.

## CRediT authorship contribution statement

**Charlotte Sahlén Helmer:** Writing – review & editing, Writing – original draft, Visualization, Validation, Software, Resources, Project administration, Methodology, Investigation, Funding acquisition, Formal analysis, Data curation, Conceptualization. **Ulrika Birberg Thornberg:** Writing – review & editing, Writing – original draft, Visualization, Validation, Supervision, Resources, Project administration, Methodology, Investigation, Formal analysis, Data curation, Conceptualization. **Thomas Abrahamsson:** Writing – review & editing, Validation, Supervision, Methodology, Investigation, Formal analysis, Data curation, Conceptualization. **Evalotte Mörelius:** Writing – review & editing, Writing – original draft, Visualization, Validation, Supervision, Software, Resources, Project administration, Methodology, Investigation, Funding acquisition, Formal analysis, Data curation, Conceptualization.

## Declaration of competing interest

The authors declare that they have no known competing financial interests or personal relationships that could have appeared to influence the work reported in this paper.

## Appendix Supplementary materials

Image, application 1

Image, application 2
