# Supplementary material for: Impacts of the Early Collaborative Intervention on mother-preterm infant interaction at one month of age: Secondary analysis of a randomized controlled trial
Source: Int J Nurs Stud Adv. 2026 Feb 9;10:100507. doi: 10.1016/j.ijnsa.2026.100507 (PMC12954294; doi:10.1016/j.ijnsa.2026.100507)
Supplement: Supplementary file 1 [file mmc1.docx]

## Supplemental material

Supplemental table 1. Intention to treat (ITT) analysis of the videotaped interaction scored with **Ainsworth***’***s Maternal Sensitivity Scales and Emotional Availability Scale direct score**.

|  | **Early Collaborative Intervention, ITT**  **n=60** | **Standard**  **care**  **n=41** | **P-value*** | **Cohen***’***s d** | **Confidence Interval**  **95% for Cohen***’***s d** | |
| --- | --- | --- | --- | --- | --- | --- |
|  |  |  |  |  | **Lower** | **Upper** |
| ***Ainsworth****’****s Maternal***  ***Sensitivity Scales,***  ***mean (SD)*** | | | | | | |
| Sensitivity | 6.12 (2.08) | 5.78 (2.30) | 0.447 | .155 | -.243 | .552 |
| Cooperation | 6.07 (1.86) | 5.68 (2.13) | 0.340 | .194 | -.204 | .592 |
| Availability | 6.98 (2.23) | 6.29 (2.56) | 0.153 | .292 | -.108 | .690 |
| Acceptance | 7.68 (1.49) | 7.22 (1.59) | 0.138 | .303 | -.097 | .702 |
| ***Emotional Availability***  ***Scales,***  ***mean (SD)*** | | | | | | |
| Sensitivity | 4.98 (1.47) | 4.89 (1.51) | 0.779 | .057 | -.340 | .454 |
| Structuring | 4.64 (1.73) | 4.50 (1.62) | 0.679 | .084 | -.314 | .481 |
| Non-intrusiveness | 4.51 (1.41) | 4.43 (1.48) | 0.759 | .062 | -.335 | .460 |
| Non-hostility | 6.34 (1.02) | 6.12 (0.99) | 0.287 | .217 | -.182 | .615 |
| Child responsiveness | 4.41 (1.60) | 4.41 (1.66) | 0.985 | .004 | -.401 | .393 |
| Child involvement | 4.03 (1.55) | 4.06 (1.74) | 0.934 | .017 | -.414 | .380 |

******Student’s t-test was used to compare means*
