# Supplementary material for: Impacts of the Early Collaborative Intervention on mother-preterm infant interaction at one month of age: Secondary analysis of a randomized controlled trial
Source: Int J Nurs Stud Adv. 2026 Feb 9;10:100507. doi: 10.1016/j.ijnsa.2026.100507 (PMC12954294; doi:10.1016/j.ijnsa.2026.100507)
Supplement: Supplementary file 2 [file mmc2.docx]

Supplemental Table 2. A comparison of demographics and characteristics of infants and mothers available at follow up and of infants and mothers not available at follow up

|  | **Available at**  **follow-up**  n=101 | **Lost at**  **follow-up**  n=42 | **P-value***** |  |
| --- | --- | --- | --- | --- |
| *Infant demographics/characteristics* |  |  |  |  |
| Gender, girls/boys | 46/55 | 16/26 | 0.413 |  |
| Twins, yes, (%) | 9, (8.9) | 6, (14.2) | 0.339 |  |
| Gestational week at birth, mean week+days, (SD) | 33+5, (1.49) | 33+2, (1.75) | 0.134 |  |
| Birthweight, gram, mean, (SD) | 2330, (529) | 2180, (617) | 0.143 |  |
| Small for gestational age, (%) | 14, (13.9) | 10, (23.8) | 0.147 |  |
| Vaginally delivery, (%) | 69, (68.3) | 26, (61.9) | 0.574 |  |
| Siblings, yes, (%)/ missing data | 45, (44.6)/ 6 | 22, (52.4)/ 2 | 0.538 |  |
| Respiratory support first week, ventilator,  CPAP, high flow, numbers, (%) | 32, (31.7) | 15, (35.7) | 0.668 |  |
| Apgar score 10 minutes, mean, (SD) | 9.64, (1.03) | 9.50, (0.83) | 0.423 |  |
| Phototherapy, numbers, (%) | 60, (59,4) | 24, (57.1) | 0.802 |  |
| Antibiotics during NICU stay, numbers, (%) | 11, (10.9) | 7, (16.7) | 0.343 |  |
| Level 3 NICU/level 2 NICU/both | 87/9/5 | 37/0/5 | 0.108 |  |
| *Maternal demographics* |  |  |  |  |
| Single parent | 2 | 3 | 0.126 |  |
| Mother*’*s age years, mean, (SD) | 31, (5.68) | 30, (5.29) | 0.153 |  |
| Maternal educational level, elementary school/high school/ university/other/missing | 1/34/53/5/8 | 0/8/10/0/24 | 0.702 |  |
| Maternal illness, numbers, (%) | 21, (20.8) | 16, (38.0) | 0.012 |  |

CPAP= Continuous Positive Airway Pressure, NICU= Neonatal Intensive Care Unit

**Student’s t-test was used to compare means. Chi-2 was used for categorical variables.*
